# A 43-year-old female with personality changes and alien limb

**DOI:** 10.1093/omcr/omaf038

**Published:** 2025-05-28

**Authors:** Anza Zahid, Zbigniew K Wszolek, Abdul R Alchaki

**Affiliations:** Stanley Appel Department of Neurology, Houston Methodist Hospital, Houston, TX, United States; Department of Neurology, Mayo Clinic, Orlando, FL, United States; Stanley Appel Department of Neurology, Houston Methodist Hospital, Houston, TX, United States; Division of Neuroimmunology, Stanley Appel Department of Neurology, Houston Methodist Hospital, Houston, TX, United States

**Keywords:** neurology, immunology, pharmacology and pharmacy

## Abstract

Colony stimulating factor-1 receptor *(CSF1R)* encodes for a tyrosine kinase receptor expressed on microglia. *CSF1R* related disorder is a devastating autosomal dominant leukoencephalopathy caused by *CSF1R* with variable penetrance in adults. It remains significantly underdiagnosed or misdiagnosed. We report a case of a 43-year-old woman with an insidious onset of neurocognitive decline, alien limb phenomenon, and personality changes over 1 year. In this report we will discuss the clinical approach, differential diagnosis, investigation, and available treatment options for *CSF1R* related disorder.

## Case report

A 43-year-old right-handed woman presented with left extremity loss of function and a change in personality over 1 year at the neurology outpatient clinic. After the birth of her fourth child, she began to experience frequent headaches. She also became withdrawn, forgetful, and volatile in her mood—crying often and laughing inappropriately. She did not have a family history of neuropsychiatric or memory disorders.

On the MoCA, she scored 13 points out of 30, losing points in visual–spatial testing, delayed memory, and calculation. Neurological exam revealed oculomotor apraxia, 3 beats of nystagmus on horizontal right-sided gaze. Grasp reflex was present bilaterally. She demonstrated significant in-coordination of her left hand, stating *‘her left hand has a mind of it’s own’.* She had increased tone in all her extremities with an admixture of rigidity and spasticity on the left side but her. Motor strength was normal. Deep tendon reflexes were exaggerated bilaterally. Sensory testing was normal. On gait assessment, she walked unassisted dragging of her left foot in planter flexed position. Romberg’s test was negative.

**Figure 1:**
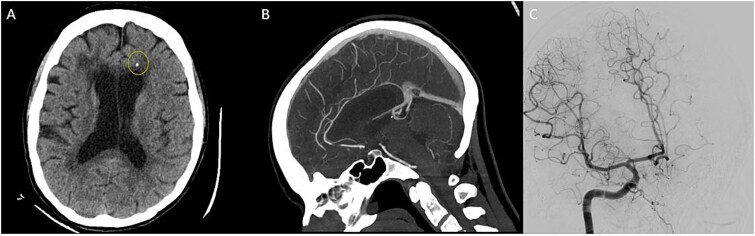
CT head showing punctate calcification in the frontal lobe (A). CT angiogram without evidence of occlusion or venous thrombosis (B). Cerebral angiogram of R carotid artery was unremarkable without evidence of beading (C).

**Figure 2:**
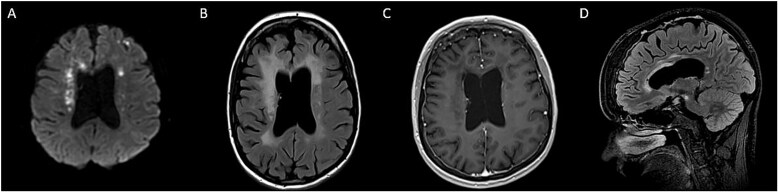
Brain MRI showing diffusion restriction (A) with T2 FLAIR confluent periventricular hyperintensity, ventricular enlargement due to frontoparietal atrophy (B) and atrophy of genu and anterior body of the corpus callosum (D) without enhancement (C).

Head CT showed ventriculomegaly and punctate calcification in the frontal lobe (Fig. 1A). Brain MRI showed diffusion restriction, T2 periventricular hyperintensity with atrophy of the genu and anterior body of the corpus callosum (Fig. 2). Cerebrospinal Fluid (CSF) revealed WBC 2/CMM, RBC 435/CMM, Protein 48 mg/dL, Glucose 62 mg/dL (serum glucose 80 mg/dl), a normal IgG index and synthetic rate.

Based on patients’ history of headaches, left-sided weakness, cognitive and personality changes differential diagnosis include cerebral venous sinus thrombosis, vascular etiology (stroke, Cerebral Autosomal Dominant Arteriopathy with Subcortical Infarcts and Leukoencephalopathy (CADASIL), Susac’s disease), neurodegenerative conditions such as behavioral variant of Fronto-temporal dementia, Corticobasal degeneration, post-COVID demyelination syndrome, or multiple sclerosis.

Frontal brain biopsy revealed extensive myelin loss with macrophage infiltration and presence of neuroaxonal spheroids. The genetic testing confirmed the diagnosis that was positive for heterozygous *CSF1R* c.1765G > A, (p.Gly589Arg) gene mutation. No pathogenic variant for NOTCH3 gene was detected. Therefore, a diagnosis of *CSF1R*-related disorder was confirmed. (Dulski J, et al Parkinsonism Related Disorders, 2024).

She was given a trial of high dose steroids without significant improvement. Induction dose of intravenous immunoglobulin (2 g/kg) showed transient benefit per oral report. For left upper extremity spasticity she was offered botulinum toxin injections.

## Discussion

Genetic testing was positive for a heterozygous mutation in *CSF1R* c.1765G > A, (p.Gly589Arg) that confirmed the diagnosis for *CSF1R-*related disorder.

*CSF1R*-related disorder is an autosomal dominant leukoencephalopathy with variable penetrance. One third to half cases are sporadic due to novel mutation and genetic mosaicism [1]. *CSF1R* encodes colony stimulating factor 1 receptor, a tyrosine kinase receptor expressed on microglia that was first identified in a cluster of families presenting with adult-onset leukoencephalopathy. Almost all mutations are located in the tyrosine kinase domain (TKD) of the *CSF1R.* Thus, the activation of *CSF1R* through autophosphorylation is required for signal transductions, which contributes to microglial maintenance and activation [2]. Advances in molecular genetics and the discovery of causative genes have facilitated increasing recognition of the disease.

The mean age at onset of *CSF1R*-related disorder is 43 years [3]. Women are observed to develop the disease 7 years earlier than men [3]. As observed in our patient, the disease is clinically characterized neuropsychiatric symptoms and parkinsonism [2].

Typical radiographic findings in *CSF1R*-related disorder includes scattered subcortical and periventricular calcifications with ‘stapping stone’ appearance on Head CT, patchy to confluent white matter abnormalities in the frontal and parietal lobes without gadolinium enhancement (Fig. 2) [3]. U-fibers are usually preserved. Thinning of corpus callosum with enlargement of ventricle and persistent diffusion restriction. [4] Subcortical high DWI signals in *CSF1R-*related strongly corelate with pathological spongiotic changes, primary axonal degeneration and loss of myelin sheaths [5]. The persistence is also hypothesized to reflect persistent intramyelinic edema in regions of neurodegeneration [5, 6]. Our patients imaging did not reveal classic radiographic features observed in *CSF1R* related disorders. She only had one small calcification without ‘stapping stone’ appearance of calcification in classic cases. Thus, brain biopsy was considered.

Historically, these patients have been misdiagnosed with multiple sclerosis or demyelinating disease, Cerebral Autosomal Dominant Arteriopathy with Subcortical Infarcts and Leukoencephalopathy (CADASIL), primary angiitis, Alzheimer Disease and fronto-temporal dementia. [3, 7] However, varying clinical and radiographic features can help differentiate *CSF1R*-related disorders from other disorders.

The neuropsychiatric symptoms manifest as a progressive decline in memory, depression, apathy, irritability, and behavior changes are observed in behavioral variant frontotemporal dementia (bvFTD). The imaging of our patient did not demonstrate typical fronto-temporal atrophy to be suggestive of bvFTD. Similarly, CADASIL can present with symptoms of unilateral weakness, dementia, migraine headaches. The classic imaging findings in CADASIL include hyperintense white matter signal abnormalities in the anterior temporal poles, centrum semi-ovale, external capsule, basal ganglia and pons along with evidence for acute infarcts and micro-hemorrhages. It is associated with a genetic mutation in *NOTCH3* gene that was negative in our patient. Cerebral angiogram was unremarkable for findings suggestive of a primary angiitis.

Similarly, both *CSF1R-* and *AARS2-*related leukoencephalopathy share several neurological symptoms and can present with similar white matter involvement, predominantly in the frontoparietal and periventricular regions [8]. However, the differences in radiological images between the two gene encoding mutations have been identified in the corpus collosum, in the regions with severe brain atrophy and in patients with *AARS2* gene mutations lack the unique calcifications that are seen on the computed tomography (CT). Unlike *CSF1R*, the *AARS2*-related phenotypes are also observed in adolescence [8].

Pathology may support the diagnosis with the presence of spheroid and pigmented glia, however, there have been reports of identical pathology in *AARS2-*related disorder without the pathogenic mutation. Hence, genetic testing is the most specific test to confirm the diagnosis [2]. (https://doi.org/10.5061/dryad.498j63f).

Unfortunately, there is no cure for *CFS1R*-related disorder. Steroids, cyclophosphamide, interferons B1a/1b, and plasmapheresis are the mainstay of treatment [9]. Symptomatic treatment and multidisciplinary approach with anti-depressants, antipsychotics, botulinum toxin injections for spasticity, rehabilitation, and referral to genetic counselling are offered. [2]

Allogenic hematopoietic stems cell transplantation (HSCT) from Human Leukocyte Antigen (HLA) matched wild-type *CSF1R* donors may offer a potential regenerative strategy in slowing neurological progression and extending survival of the patients to about 15 years in selected cases [10]. Acute or chronic graft versus host disease was not observed with HSCT, however, worsening neurological symptoms including; extrapyramidal symptoms, parkinsonism, pneumonia, and new localization-related seizures has been reported [10].

IGNITE Phase 2 trial of Iluzanebart (VGL101) using TREM2 agonist is showing promise at 6-month assessment in patients with CSF1R-related disorder. Iluzanebart (VGL101) is a humanized monoclonal antibody that binds with TREM2 to activate the signaling pathway to increase the ability of microglia and to protect the neurons from damage (NCT05677659). [1, 2]

Early genetic testing is critical in patients with possible CSF1R related disorder to enable early treatment and slow disease progression [2].

### Patient outcome

Our patient’s disease had progressed too far, rendering her ineligible for clinical trials. She was given a trial of high dose steroids without significant improvement. Induction dose of intravenous immunoglobulin (2 g/kg) showed transient benefit per oral report. For left upper extremity spasticity she was offered botulinum toxin injections. The family was referred to a genetic clinic for counseling for her 4 children.
